# Secondary formation damage of low-pressure layer during commingled production in multilayered tight gas reservoirs

**DOI:** 10.1038/s41598-019-53940-6

**Published:** 2019-11-26

**Authors:** Jingchen Ding, Changhui Yan, Yongming He, Changcheng Wang

**Affiliations:** 1 Post-Doctor Station, Chengdu University of Technology, Chengdu, China; 2 Exploration and Development Research Institute, SINOPEC North China Company, Zhengzhou, China

**Keywords:** Energy science and technology, Engineering

## Abstract

This paper experimentally investigates fluid back-flow behavior and formation damage during commingled production in multilayered tight gas reservoirs. The development of fluid back-flow in commingled tight gas reservoirs was simulated using a newly designed experimental platform. The results indicate that when there is a pressure difference between different layers during commingled production from tight gas reservoir, water produced from the high-pressure layer will invade the low-pressure layer along with gas back-flow and will accumulate in the near-wellbore area. This will lead to an increase in water saturation and a decline in permeability in the low-pressure layer and result in a significant reduction in ultimate recovery. The outcomes of these experiments demonstrate that as well as the formation damage caused by the working fluid during drilling and fracturing, “Secondary Formation Damage” also occurs during commingled production in multilayered tight gas reservoirs. This secondary formation damage mainly occurs in the near-wellbore area of low-pressure layers and is more severe with greater proximity to the wellbore. Through further experimentation to assess the factors influencing secondary formation damage, it is shown that the degree of secondary formation damage increases with decreasing original formation pressure, original water saturation, and permeability in the lower-pressure layer.

## Introduction

Natural gas is being developed at an ever-increasing rate worldwide due to its environmental benefits relative to other fossil fuels. Tight gas, which is a major emerging source of natural gas supply, has entered a “golden age” driven by the decline of conventional natural gas resources^1–3^. In China, for example, tight gas production reached 51.5 billion cubic meters in 2018, 32 percent of the national natural gas production. It is anticipated that, by 2020, tight gas production will rise to 80 billion cubic meters and will account for up to 36 percent of China’s natural gas supply, according to the National Energy Administration of China^4,5^.

Tight gas reservoirs are commonly thin and have multiple development layers. Single-layer development often fails to meet economic requirements due to the poor reservoir properties and insufficient thickness offered by a single layer^6^. Therefore, a considerable proportion of tight gas reservoirs adopt the development method of commingled production from vertical wells^7–9^. However, since different layers always have different reservoir and fluid properties, interlayer interference is inevitable in commingled production^10,11^. Interlayer interference has thus become a topic of considerable interest in tight gas research, and extensive work has been done in this area^12–16^.

Interlayer fluid exchange through a wellbore is one of the main mechanisms of interlayer interference. Fluid crossflow can occur from one layer to another when there is a pressure difference between different layers, and this can affect the whole commingled production process^17,18^. Zhu and Hill (2002) developed a multilateral well deliverability model that couples reservoir inflow into multiple well segments and found that crossflow from a lower to an upper layer can occur in multilateral wells if the surface pressure is too high^11^. Hong *et al*. (2012) reported that the interference coefficient is positively correlated with the permeability contrast and also found that interlayer interference decreases gradually as multilayer commingled production continues^19^. Zhu *et al*. (2013) carried out a commingled production experiment on a multilayer gas reservoir and demonstrated that back-flow from high to low-pressure layers can occur easily and may cause interlayer pressure fluctuations and related reservoir damage^20^. A quantitative evaluation of the degree of interference in dual-reservoir commingled production of tight sand gas in the Linxing area by Feng *et al*. (2017) showed that fluid back-flow will lead to a production decline during the early development stages but has little impact on long-term development^21^.

There is now a general consensus that back-flow damage during commingled production is temporary and will not have any impact on long-term production or ultimate recovery levels^20,22–24^. However, development data from some tight gas reservoirs shows that during commingled production, the low-pressure layers of a partial gas well show a significant permeability decline in the near-wellbore area after fluid back-flow, with a corresponding significant drop in productive capacity. The studies discussed above cannot explain this observation. Meanwhile, some researchers have also come to realize that interlayer fluid exchange during commingled production is not only of gas; water may also enter a low-pressure layer along with back-flow gas and thus may cause reservoir damage and a decline in productivity^21^. However, until now, no experiments have been reported on two-phase (gas and water) back-flow in commingled production in multilayered tight gas reservoirs, hindering an understanding of the potential for reservoir damage in this production context. It remains to be explored whether water will back-flow into the low-pressure layer, and, if so, whether the invading water will affect the physical properties of and recovery from that layer. It must further be determined whether the impact of this back-flow will be different under different reservoir combinations.

In this study, experimental investigations were first performed to study two-phase back-flow and reservoir damage during commingled production in multilayered tight gas reservoirs. The formation and development of two-phase back-flow were simulated using a newly developed procedure, and the concept of “Secondary Formation Damage” that occurs during commingled production was proposed. On this basis, the mechanism, range of influence and degree of influence of secondary formation damage were experimentally investigated, and the factors influencing secondary formation damage were explored. This study investigates reservoir damage development during commingled production and contributes to our present understanding of commingled production in water-bearing multilayered tight gas reservoirs.

## Materials and Conditions

### Cores

To simulate commingled production from tight gas reservoirs more accurately, experiments were carried out on natural cores from the DS gas reservoir, a tight sand gas reservoir in western China. The rock type is moderately sorted lithic-quartz sandstone.

Short natural cores with similar permeability values were connected in series to form a long core to simulate the underground gas layers. This use of long cores enables better analysis of the radial distribution of pressure and fluid during commingled production than would a more limited core. The lengths of the individual short cores range from 6.3 cm to 6.8 cm. Each long core consists of 15 short cores and has a total length of about 100 cm. Five long cores with average permeability range from 0.11 × 10^−3^ μm^2^ to 2.11 × 10^−3^ μm^2^ were prepared; their properties are shown in Table 1.

**Table 1:** Properties of long cores.

Name	Average Permeability, 10^−3^ μm^2^	Porosity, %	Diameter, cm	Total Length, cm
Long Core I	0.11	7.5	2.5	98
Long Core II	0.23	8.7	2.5	100
Long Core III	0.85	10.4	2.5	99
Long Core IV	2.05	12.8	2.5	100
Long Core V	2.11	12.9	2.5	99

### Fluids

The gas used in this study is pure nitrogen (99.999%). The water used was prepared indoors according to the parameters of the formation water of the DS gas reservoir, which are shown in Table 2.

**Table 2:** Parameters of the formation water of the DS gas reservoir.

PH	Ion Concentration, mg/L								Total Salinity, mg/L	Water Type
	HCO_3_^−^		Cl^−^		SO_4_^2−^	Ca^2+^	Mg^2+^	K^+^ + Na^+^		
6	113	24788		2225		7961	125	6796	42008	CaCl_2_

### Experimental conditions

Experimental conditions of this study are shown in Table 3. Experimental pressure and temperature are set to simulate the actual reservoir condition of DS tight gas reservoir (Appendix A).

**Table 3:** Experimental conditions.

Parameter	Value	Parameter in the DS Gas Reservoir Replicated
Experimental temperature	75 °C	Reservoir temperature
Confining pressure on the long core	51 MPa	Overburden pressure
Maximum initial pore pressure	27 MPa	Original reservoir pressure
Back pressure	18 MPa	—

## Experimental Equipment and Procedure

**Figure 1:**
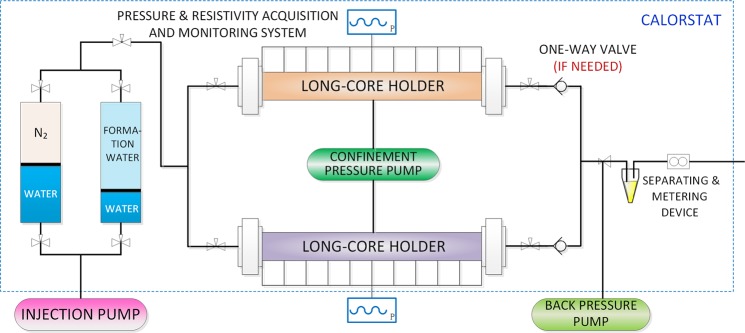
Experimental procedure to study formation damage during commingled production.

The experimental procedure is shown in Fig. 1. The prepared long core was placed in a long-core holder. A pressure sensor and resistivity sensor was installed every 10 cm along the holder, so that real-time changes in pressure and water saturation along the long core during the experiments could be recorded.

A back-pressure valve was set on the main pipeline downstream of two long-core holders. Back pressure was exerted using an ISCO pump to simulate constant-pressure production of a gas reservoir. Two long-core holders were connected in parallel by the gas production pipeline to simulate a two-layer tight gas reservoir in commingled production. In addition, the whole experimental procedure was placed in a calorstat to simulate the actual temperature of the reservoir.

The water saturation of each core was first brought to its prescribed value, and nitrogen was then injected until the pore pressure reached the pre-set value, at which point the inlet valve of the long-core holder was closed. At the beginning of each experiment, the outlet valves of the two long-core holders were opened at the same time to simulate commingled production. During the experimental process, pressure and resistivity along each long-core holder were recorded at regular intervals, and the fluid produced from the main pipeline was separated into water and gas, which were measured separately. The experimental run ended when no more fluid was being produced.

For experimental runs simulating commingled production without back-flow, a one-way valve was set in the outlet pipeline of each long-core holder. This prevented back-flow of the fluid produced into the core. For comparative experiments, the one-way valves were removed so that back-flow could occur. On the basis of these comparative experiments, the influence of back-flow on commingled production could be isolated and studied.

## Results and Discussion

### Commingled production behavior of dry cores

**Figure 2:**
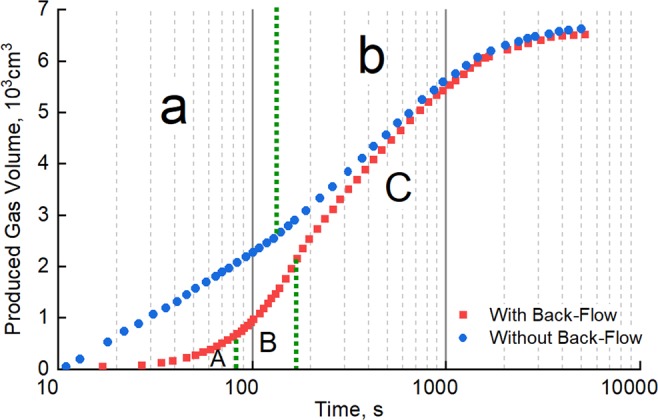
Commingled production behavior of dry cores with and without back-flow.

First, commingled production was simulated using dry cores to provide a reference frame for subsequent experiments. Long Core IV and Long Core V were selected for these experiments; their pore pressures were set at 27 MPa and 22 MPa, respectively. Commingled production simulation was carried out both with and without back-flow, giving the results shown in Fig. 2.

Without back-flow, the total gas production curve for commingled production exhibits two stages. During early production (stage a), gas produced from the high-pressure layer (Long Core IV) is at higher pressure than that from the low-pressure layer (Long Core V). Gas cannot be produced from the low-pressure layer, and all of the gas is supplied from the high-pressure layer. Gas in the low-pressure layer begins to be released when the outlet pressure of the commingled production is lower than the pressure in the low-pressure layer, resulting in an increase in the gas production rate (stage b). As the gas is produced continuously from two layers, the total gas production rate gradually decreases and tends to stabilize.

Taking back-flow into account results in a significantly different gas production curve that reflects three-stage gas-production behavior. In stage A, which we call the “initial stage of back-flow”, a large proportion of the gas produced from the high-pressure layer flows back into the low-pressure layer, leading to a significant decrease in total gas production versus the case without backflow. When commingled production enters stage B, which we call the “recovery stage of back-flow”, the invading gas in the low-pressure layer begins to be re-produced as the outlet pressure of commingled production decreases, and there is a rapid increase in total gas production. During stage C, which we call the “commingled production stage”, the effects of back-flow gradually disappear, and the total gas production behavior tends to be consistent with that without back-flow.

At the end of these experimental runs, total production in the case of back-flow is slightly lower than that without back-flow. These results agree with former studies that indicate that commingled production has little impact on long-term gas recovery^20,22^.

**Figure 3:**
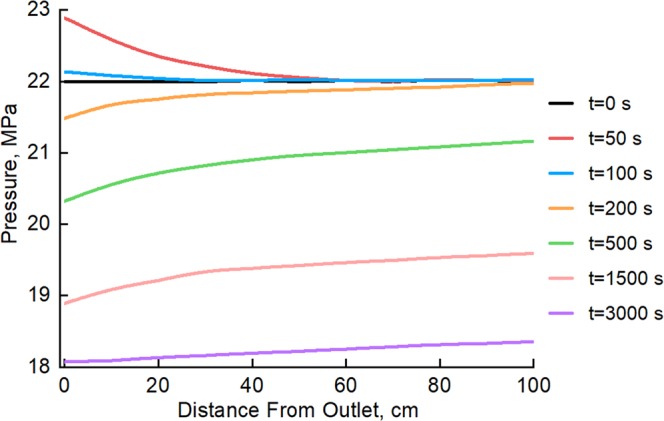
Pressure distribution along the low-pressure layer during commingled production (with back-flow).

The pressure distribution along the low-pressure layer during commingled production with backflow, which is shown in Fig. 3, more clearly illustrates the back-flow process. Pore pressure near the outlet of the low-pressure layer shows a significant increase instead of a decrease at the very beginning of commingled production (t = 50 s). The pressure increase is greater at closer proximity to the outlet. This is the behavior one would intuitively expect with fluid back-flow during commingled production; this time corresponds to the initial stage of back-flow in Fig. 2. When the time elapsed reaches 100 s, the pressure increase near the outlet is significantly reduced. This is because the fluid that has invaded begins to be re-produced as the outlet pressure of commingled production decreases; this time corresponds to the recovery stage of back-flow in Fig. 2. The overall pressure of the low-pressure layer then begins to decline gradually, which indicates that the original fluid in the low-pressure layer has begun to be produced, corresponding to the commingled production stage in Fig. 2.

The changes in the pressure distribution along the low-pressure layer demonstrate that the back-flow has a sphere of influence of 50–60 cm. The section of the core far from the outlet shows no pressure perturbation during the back-flow stage. These results reveal that the impact of back-flow is limited to the near-wellbore area of the lower-pressure layer, and back-flow has little effect on the remote formation.

### Commingled production behavior and secondary formation damage of water-bearing cores

**Figure 4:**
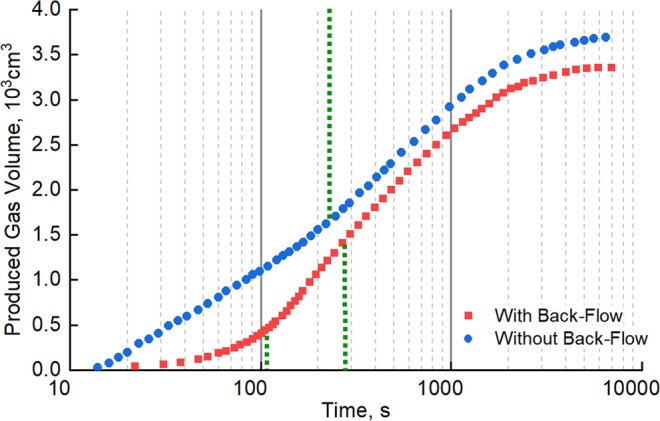
Commingled production behavior of water-bearing cores with and without back-flow.

The results above confirm previous findings on the commingled production behavior of dry cores. However, gas–water two-phase flow is ubiquitous in most tight gas reservoirs, so further experiments were carried out on Long Cores IV and V to explore their behavior under water-saturated conditions^25–27^. The water saturation values of the long cores were set at 55% and 20% respectively to simulate the reservoir conditions. The other experimental conditions and experimental procedures were the same as in Section 4.1. The results are shown in Fig. 4.

The total gas production curves for commingled production with water-bearing cores show similar trends to those for dry cores. However, the final gas production volume in the case with back-flow is significantly lower than that without back-flow, unlike with dry cores.

**Figure 5:**
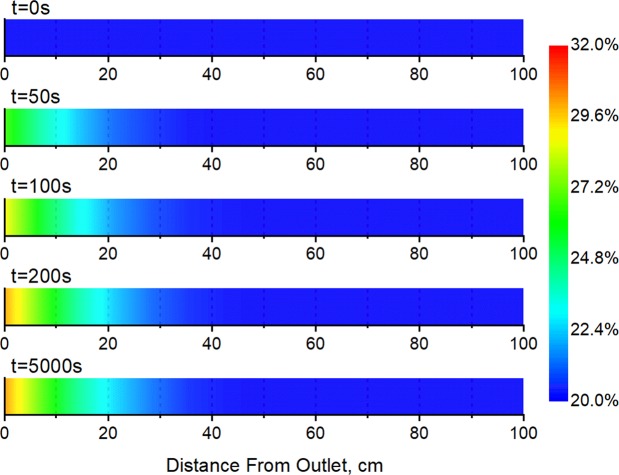
Change in water saturation along the low-pressure layer during commingled production (with back-flow).

The reasons behind this difference in total production were explored by studying the change in water saturation along the low-pressure layer during the experimental process in the case with back-flow (Fig. 5). The water saturation near the outlet of the low-pressure layer rises as the experiment progresses. This is because the water produced from the high-pressure layer enters the low-pressure layer along with the gas back-flow and accumulates in the near-wellbore area, increasing its water saturation. Parts of the core that are far from outlet are not affected by the back-flow, so the water saturation remains constant. After the back-flow stage, the water saturation near the outlet of the low-pressure layer stabilizes, remaining significantly higher than that in other sections.

**Figure 6:**
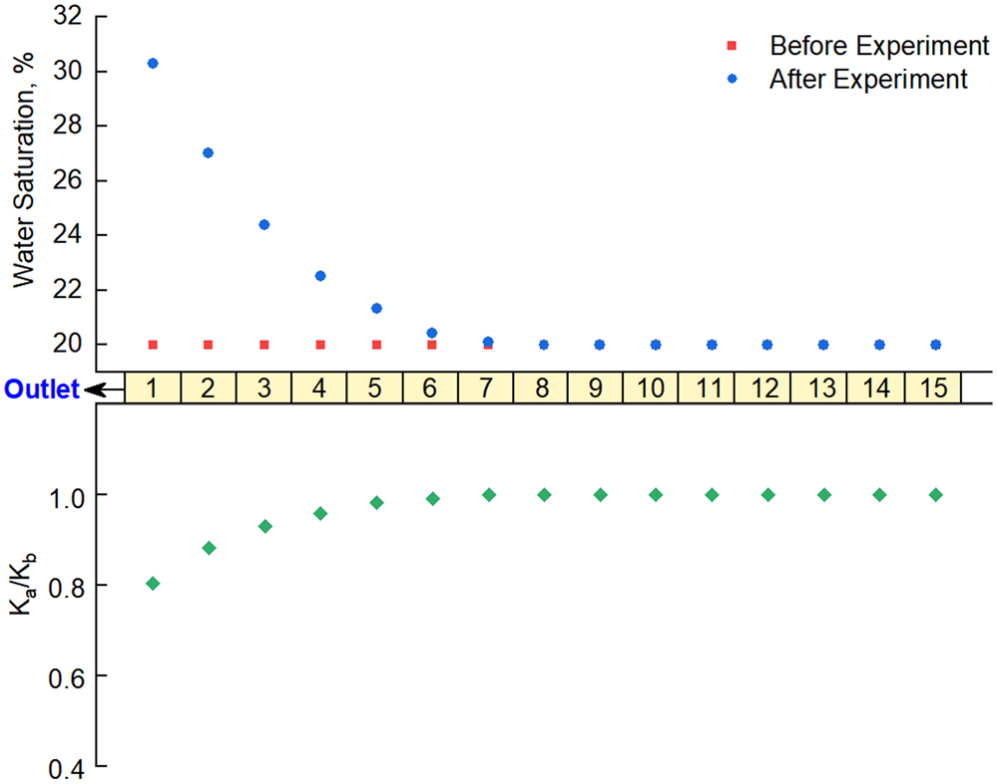
Water saturation and permeability of low-pressure-layer sub-cores before and after commingled production (with back-flow).

At the end of the experiment, the short cores making up the low-pressure layer were removed one by one, and their permeability and water saturation values were measured individually. The properties of the sub-cores before and after the experiment were compared, as shown in Fig. 6.

It is clear from Fig. 6 that sub-cores near the outlet of the low-pressure layer were affected significantly by the back-flow and show increased water saturation after the experiment. The closer they were to the outlet, the more their water saturation had increased. Sub-cores that were far from the outlet were not affected by the back-flow: their water saturation had not changed after the experiment.

The results for the ratio between sub-core permeability before and after the experiment, K_a_/K_b_, plotted in Fig. 6, show that the permeability of sub-cores near the outlet of the low-pressure layer had declined by 19.5% after the experiment. Moreover, the gas relative permeability showed a decline of 25.7% according to the rock relative permeability data. This is mainly because water invaded the low-pressure layer along with the gas back-flow and will have led to an increase in water saturation and to water block^28–30^. The closer they were to the outlet, the greater the influence of the back-flow, the greater the increase in water saturation, and the greater the decline in permeability.

These results suggest that during commingled production from a tight gas reservoir, water produced from the high-pressure layer will enter the low-pressure layer along with back-flowing gas, and this will lead to an increase in water saturation and a decline in permeability and gas relative permeability in regions near the wellbore, resulting in a reduction in the seepage capability of the low-pressure layer. This is the reason why the final gas production volume in the case with back-flow is significantly lower than that without back-flow under commingled production from water-bearing cores (Fig. 4).

Unlike the formation damage caused by working fluid^31,32^, the damage found in this study occurs during commingled production from gas reservoirs and is caused by back-flow of the produced formation fluid and its invasion into a low-pressure layer. Therefore, we term this damage “Secondary Formation Damage”.

Next, a series of experiments were carried out to study the factors influencing this secondary formation damage.

### Secondary formation damage under different initial low-pressure-layer pore pressures

**Figure 7:**
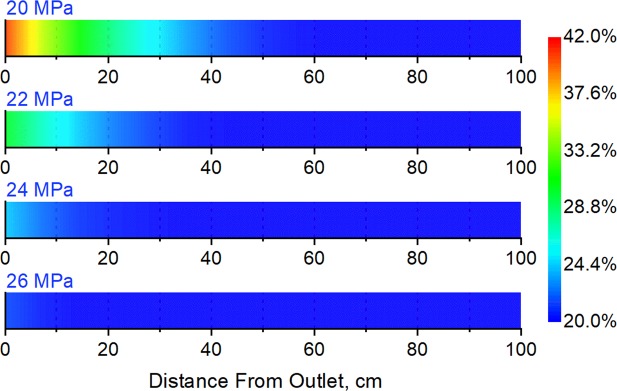
Water saturation of the low-pressure layer after commingled production under different initial low-pressure-layer pore pressures.

**Figure 8:**
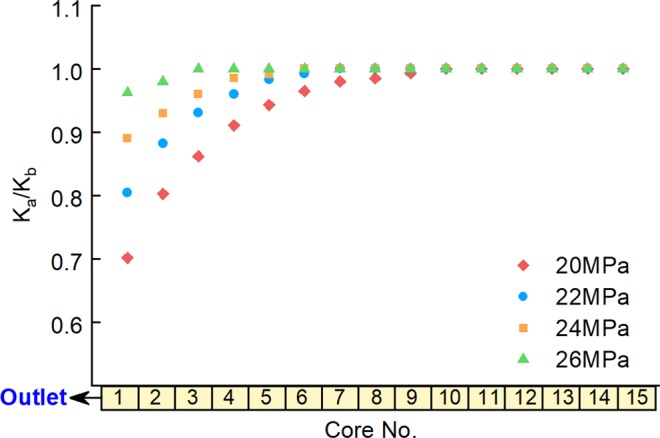
Ratio between the permeability of low-pressure-layer sub-cores before and after commingled production under different initial low-pressure-layer pore pressures.

The influence of the initial pore pressure of the low-pressure layer was investigated by conducting experiments in which Long Core IV, the high-pressure layer, had an initial pore pressure of 27 MPa, and Long Core V, the low-pressure layer, had an initial pore pressure of 20 MPa, 22 MPa, 24 MPa, and 26 MPa in different experimental runs. The water saturation values of the high and low-pressure layers were set at 55% and 20%, respectively. Experiments were carried out with back-flow, with the results shown in Figs. 7 and 8.

The figures show that back-flow and secondary formation damage have little influence on the low-pressure layer when its initial pore pressure is close to that of the high-pressure layer (where it is 26 MPa). The water saturation is almost the same after the experiment as it was before it, and there her been a slight decrease in the permeability of sub-cores near the outlet. Secondary formation damage becomes more obvious as the initial pore pressure of the low-pressure layer decreases: the water saturation and permeability of sub-cores near the outlet show a significant increase and decrease after the experiments, respectively. The lower the initial pore pressure of the low-pressure layer, the larger the range and degree of influence of secondary formation damage become.

Furthermore, additional experiments were performed under the same conditions as for the four experimental runs described above but by setting the one-way valve at the outlet pipeline of each long-core holder, thus preventing back-flow and avoiding secondary formation damage. The gas recoveries at different initial pore pressures in the low-pressure layer without and without secondary formation damage were compared, as shown in Table 4.

**Table 4:** Influence of secondary formation damage on gas recovery for different initial low-pressure-layer pore pressures

Initial Pore Pressure of Low-Pressure Layer, MPa	20	22	24	26
Gas Recovery Decline, %	14.7	9.7	6.5	4.2

Table 4 shows that gas recovery was always lower when secondary formation damage was taken into account than when it was not. The influence of secondary formation damage on gas recovery increases as the initial pore pressure of the low-pressure layer decreases. These findings can be attributed to the increase in the range and degree of influence of secondary formation damage with a decrease in the initial pore pressure of the low-pressure layer, which results in permeability loss and will finally lead to the recovery decline.

### Secondary formation damage under different low-pressure-layer permeability values

**Figure 9:**
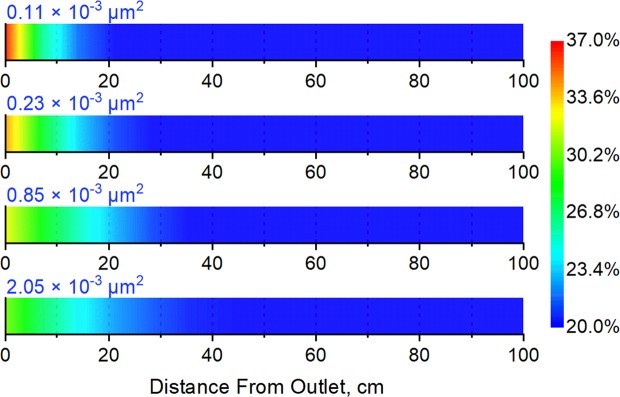
Water saturation of the low-pressure layer after commingled production for different low-pressure-layer permeability values.

**Figure 10:**
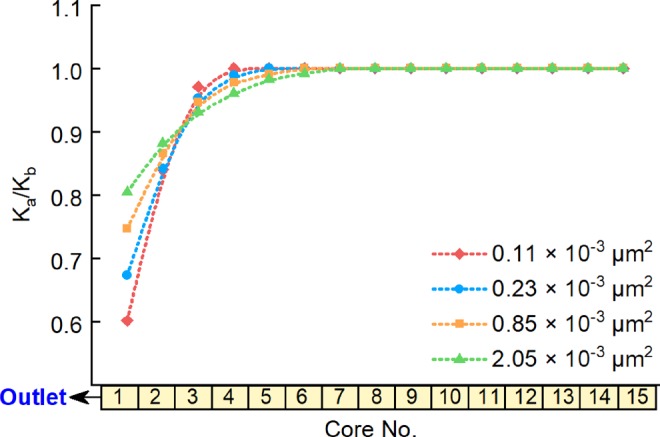
Ratio between the permeability of low-pressure-layer sub-cores of the before and after commingled production for different low-pressure layer permeability values.

To investigate the influence of the permeability of the low-pressure layer on secondary formation damage, Long Core V was set as the high-pressure layer, with an initial pore pressure of 27 MPa and a water saturation of 55%. Long Core I, Long Core II, Long Core III, and Long Core IV, which have different average permeability values (Table 1), were set as the low-pressure layer for different experimental runs. The low-pressure layer had an initial pore pressure of 22 MPa and a water saturation of 20%. The results of experimental runs simulating commingled production under these conditions are shown in Figs. 9 and 10.

The figures show that the range of influence of secondary formation damage decreases with a decrease in the permeability of the low-pressure layer. However, within its sphere of influence, the increase in water saturation and the decrease in permeability are greater at lower low-pressure-layer permeability values. This is mainly because lower permeability makes it more difficult for the invading fluid to enter the low-pressure layer, which will shorten the range of influence of secondary formation damage.

At the same time, however, water imbibition and the water block effect increase considerably in case of low permeability, which will lead to stronger secondary formation damage. According to the water saturation change, the volume of water invading the low-pressure layer was 0.61 cm^3^, 0.78 cm^3^, 1.02 cm^3^ and 1.10 cm^3^ respectively in case of the permeability of low-pressure layer ranged from 0.11 × 10^−3^ μm^2^ to 2.01 × 10^−3^ μm^2^. The lower the permeability of the low-pressure layer, the less the amount of invasion water. However, since the low-permeability layer has lower porosity, and the range of invasion water decreases with the decreasing permeability. Therefore, although the total amount of water invaded through back-flow reduces in the case of low permeability, the influence of the invasion water on the seepage capacity of the near-wellbore area still increases.

As described in Section 4.3, experiments were performed without secondary formation damage (without back-flow), and the gas recoveries were compared with those from experiments with secondary formation damage; the results are shown in Table 5.

**Table 5:** Influence of secondary formation damage on gas recovery for different low-pressure-layer permeability values.

Permeability of Low-Pressure Layer, ×10^−3^ μm^2^	0.11	0.23	0.85	1.99
Gas Recovery Decline, %	18.7	14.8	12.1	9.7

The influence of secondary formation damage on gas recovery can be seen to increase with a decrease in the permeability of the low-pressure layer. As shown by the results above, although a decrease in the permeability of the low-pressure layer does lead to a reduction in the range of the secondary damage, it also leads to a substantial increase in its degree of influence; hence, recovery is increasingly severely impacted.

### Secondary formation damage under different initial low-pressure-layer water saturation values

**Figure 11:**
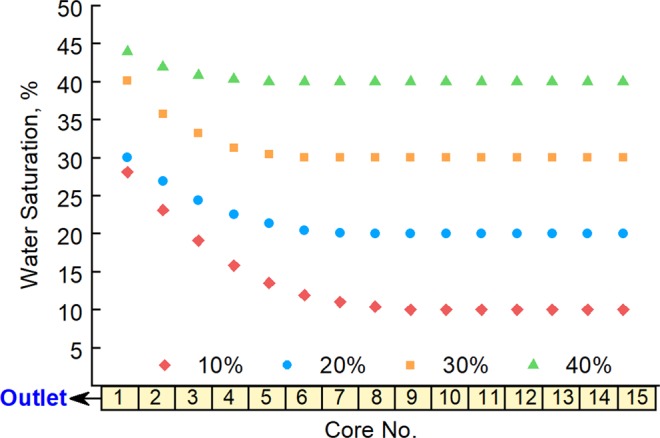
Water saturation of the low-pressure layer after commingled production for different initial low-pressure-layer water saturation values.

**Figure 12:**
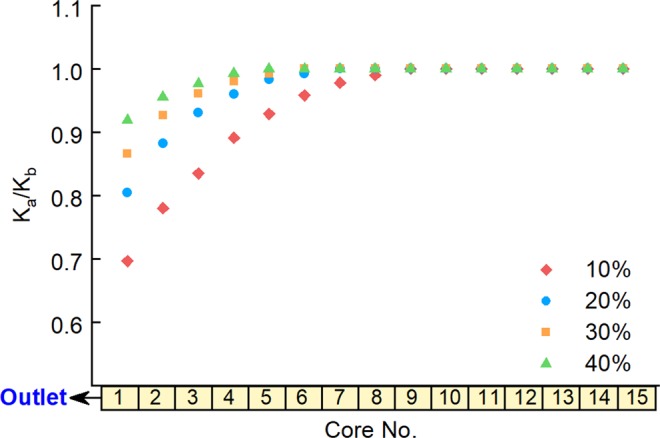
Ratio between the permeability of low-pressure-layer sub-cores before and after commingled production with different initial low-pressure-layer water saturation values.

To explore the influence of the initial water saturation of the low-pressure layer, further experiments were performed using Long Core IV as the high-pressure layer, with an initial pore pressure of 27 MPa and water saturation of 55%, and Long Core V as the low-pressure layer, with a pore pressure of 22 MPa and a water saturation of 10%, 20%, 30% and 40% in different experimental runs. Experiments were carried out with back-flow, giving the results shown in Figs. 11 and 12.

The figures show that both the range and degree of influence of secondary formation damage increases with a decrease in the initial water saturation of the low-pressure layer. The permeability of sub-cores near the outlet had decreased to about 70% of the initial value after the experiment in the case with 10% initial water saturation of the low-pressure layer, whereas the permeability ratio was about 92% in the case of a 40% initial water saturation. This is mainly because flow resistance shows a marked reduction with a decrease in water saturation. Therefore, the back-flow fluid will be able to enter the low-pressure layer more easily, causing secondary formation damage to have a larger range and degree of influence.

As in the previous sections, experiments were performed without secondary formation damage (without back-flow), and the gas recovery values from these experiments were compared to those from the experiments with secondary formation damage; the results are shown in Table 6.

**Table 6:** Influence of secondary formation damage on gas recovery under different initial low-pressure-layer water saturation values

Initial Water Saturation of Low-Pressure Layer, %	10	20	30	40
Gas Recovery Decline, %	14.5	9.7	7.4	5.2

The influence of secondary formation damage on gas recovery can be seen to increase with a decrease in the initial water saturation of the low-pressure layer. This can be explained by the results above, which show that the range and degree of secondary formation damage to increase considerably with a decrease in the initial water saturation of the low-pressure layer; this will cause recovery to become correspondingly reduced to an increasingly severe degree.

## Conclusions

1. When there is a pressure difference between different layers during commingled production from a tight gas reservoir, water produced from the high-pressure layer will invade the low-pressure layer along with gas back-flow, leading to a decline in permeability. “Secondary Formation Damage” thus develops, resulting in a significant reduction in ultimate recovery.
2. Secondary formation damage occurs in the near-wellbore area of low-pressure layers and is more severe at closer proximity to the wellbore.
3. The range and degree of influence of secondary formation damage increase with decreasing initial pore pressure and water saturation in the low-pressure layer.
4. With a decrease in the permeability of the low-pressure layer, the range of influence of secondary formation damage reduces, but its degree of influence shows a considerable increase.

## Experimental Temperature

Experimental temperature was set at 75 °C according to the actual reservoir temperature of DS tight gas reservoir.

## Experimental Pressure

Confining pressure on the long core was set to simulate overburden pressure of DS tight gas reservoir. In fact, reservoirs may have different confining pressure on the upper and lower layers for commingle wells. But results from our preliminary research show that when the pore pressure is 27 MPa (original reservoir pressure of DS tight gas reservoir), the permeability differs only 5.5% under confining pressure 48 MPa and 55 MPa (overburden pressure of different layers in DS tight gas reservoir). Different confining pressures have little effect on core permeability and experimental results. Therefore, for the sake of simplicity, different layers share the same confining pressure in the experiments. The confining pressure was set at 51 MPa, average overburden pressure of different layers in DS tight gas reservoir

Initial pore pressure was set to simulate the original reservoir pressure of DS tight gas reservoir. The high-pressure layer had an initial pore pressure of 27 MPa, the same as the *in-situ* conditions. Initial pore pressure of low-pressure layer ranged from 20 MPa to 26 MPa to study the influence of pressure difference on commingled production and secondary formation damage.

Back pressure was set at 18 MPa to simulate the constant well-head pressure production.

## Water Saturation of Long-Core

### Water saturation value

Water Saturation of the high-pressure layer was set at 55%, the *in-situ* value of water-rich layer in DS tight gas reservoir. Initial water Saturation of low-pressure layer ranged from 10% to 40% to study the influence of initial water saturation on commingled production and secondary formation damage.

### Water saturation establishment

To guarantee the uniformly distribution of water saturation of the long core, for each short core, a clean-and-dry process was firstly carried out, then the core was vacuumed and saturated with simulated formation water. Use high temperature nitrogen (90 °C) to drive the core, and the core was removed from the holder and weighed every 5 minutes during the driving process. The real-time water saturation was calculated using the core weighing data. When the water saturation reached the set value, the displacement was terminated and the core was sealed and stored in a dark and low temperature environment (to avoid evaporation). After the water saturation of all short cores was established, assemble all the short cores into the long core. By using this method, water saturation of each short core in the long core can be guaranteed to a certain value and thus we can make sure the initial water saturation is uniformly distributed along the long core.
